# Evidence of decline of malaria in the general hospital of Libreville, Gabon from 2000 to 2008

**DOI:** 10.1186/1475-2875-8-300

**Published:** 2009-12-17

**Authors:** Marielle Karine Bouyou-Akotet, Denise Patricia Mawili-Mboumba, Eric Kendjo, Modeste Mabika-Mamfoumbi, Edgard Brice Ngoungou, Arnaud Dzeing-Ella, Mireille Pemba-Mihindou, Euloge Ibinga, Emmanuel Efame-Eya, MCRU team, Tim Planche, Peter G Kremsner, Maryvonne Kombila

**Affiliations:** 1 Department of Parasitology-Mycology and Tropical Medicine, Faculty of Medicine, Université des Sciences de la Santé, BP 4009 Libreville, Gabon; 2 Malaria Clinical Research Unit (MCRU), Centre Hospitalier de Libreville, Libreville, Gabon; 3 Medical Research Unit, Albert Schweitzer Hospital, Lambaréné, Gabon; 4 Institute of Tropical Medicine, University of Tübingen, Germany; 5 Department of Infectious Diseases, St George's Hospital Medical School, Cranmer Terrace, London, UK

## Abstract

**Background:**

Substantial decline in malaria transmission, morbidity and mortality has been reported in several countries where new malaria control strategies have been implemented. In Gabon, the national malaria policy changed in 2003, according to the WHO recommendations. The trend in malaria morbidity was evaluated among febrile children before and after their implementation in Libreville, the capital city of Gabon.

**Methods:**

From August 2000 to December 2008, febrile paediatric outpatients and inpatients, under 11 years of age, were screened for malaria by microscopic examination at the Malaria Clinical Research Unit (MCRU) located in the largest public hospital in Gabon. Climatic data were also collected.

**Results:**

In total, 28,092 febrile children were examined; those under five years always represented more than 70%. The proportion of malaria-positive slides was 45% in 2000, and declined to 15% in 2008. The median age of children with a positive blood smear increased from 24(15-48) to 41(21-72) months over the study period (*p *< 0.01). Rainfall patterns had no impact on the decline observed throughout the study period.

**Conclusion:**

The decrease of malaria prevalence among febrile children during the last nine years is observed following the introduction of new strategies of malaria cases management, and may announce epidemiological changes. Moreover, preventive measures must be extended to children older than five years.

## Background

Malaria has, for a long time, been the major cause of febrile illness in sub-Saharan Africa, where 80% of the 247 million cases were reported in 2006 [1]. The appearance of multidrug-resistance *Plasmodium falciparum *has led to the introduction of new control strategies. Insecticide-treated bed nets (ITNs), along with prompt and effective treatment of clinical malaria cases, intermittent preventive treatment in pregnant women, and indoor residual spraying, are now being deployed widely across Africa with increasing amounts of coverage achieved [2-4]. There is growing evidence documenting a substantial decline in malaria transmission, morbidity and mortality in several countries where these interventions have been implemented including areas with high levels of transmission [5-8]. One of the major challenges for the effective monitoring and evaluation of the impact of malaria control tools is to provide epidemiological informations in endemic areas such as Gabon. Throughout this country, infections with *P. falciparum *accounted for 30% to 50% of the consultations of children with fever between 1980s and 1990s [[9-11], Kombila et *al*, unpublished data]. Resistance to chloroquine appeared in the 1980s and rose to 100% since 2001 [12-14]. In 2003, Gabonese Ministry of Health changed the malaria national policy to follow the WHO 2000 recommendations [15]. Artemisinin based combination therapies (ACTs) were adopted as first- and second-line treatment for uncomplicated malaria, and along with ITNs and sulfadoxine-pyrimethamine for intermittent preventive treatment (IPTp-SP) were freely available in all public health centres of country since 2005, for children under five years old and pregnant women. Reduced malaria prevalence in pregnant women was observed in Libreville and Lambaréné following IPTp-SP implementation [16]. In this study, the trend in malaria morbidity was evaluated among febrile children, over a nine-year period, in the largest public hospital of Libreville, the capital city of Gabon.

## Methods

### Study site and population

This prospective and observational study was conducted from August 2000 to December 2008 in Libreville, the capital city of Gabon, where the climate is equatorial. In this urban area, malaria is predominantly caused by *P. falciparum*. The population of Libreville was estimated to be 537,540 inhabitants in 2006, with children under five years of age representing 16%.

The Malaria Clinical Research Unit (MCRU) is a branch of the Department of Parasitology of the Medicine Faculty, located in the Centre Hospitalier de Libreville (CHL), the largest public hospital in Gabon. At MCRU, all febrile paediatric outpatients and inpatients, aged less than 11 years old, were prospectively screened for malaria. In this unit, children routinely benefit for free malaria diagnosis based on microscopic examination. Temperature, history of fever, age, sex, and location were collected prospectively.

### Malaria diagnosis

Throughout the study period, a thick blood film was done for each patient. Blood slides were processed according to the Lambaréné procedure, which is described elsewhere [17]. Slides were considered negative if no asexual blood stages of parasites and gametocytes were seen in 100 oil-immersion fields. Quality control of the blood smears was performed: a second microscopist, blinded to the results of the first reading, read the slides. In case of disagreement, slides were controlled by a third microscopist and the mean of the two closest readings of parasitaemia was taken. The case definition of malaria was any febrile child with slide-confirmed parasites.

### Rainfall patterns

Monthly rainfall data of Libreville for the last decade were obtained from the meteorological services of the Agency for Air Navigation Safety in Africa and Madagascar (ASECNA).

### Data analysis

Demographic and laboratory data were recorded and given to the data manager. Data were entered and cleaned using Epi-info version 3.3.2 (February 9, 2005 CDC Atlanta) and were analysed with STATA version 10 (Stata Corp, College station, USA). Medians with interquartile ranges (IQR) of age and geometric means of parasite densities (GMPD) are presented. Differences between groups were assessed using Pearson's chi-squared or Fisher's exact tests for proportions. For ordinal outcomes, the linear trend test (Cochran-Armitage test for trend) was used. The association between age and risk of malaria by year was assessed using odds ratio and the 95% confidence interval (OR (95%CI)). Linear regression was applied to test the significance of decreases for log-transformed data. To take into account the effect of auto-correlated observations and the season disturbances, an ARMAX model was built including covariates. We postulated a straightforward relationship between proportion of malaria cases and the monthly rainfall average measured by 20-point first-order moving average that was applied to filter seasonal variation and highlight the long-term movements in the data. Deviations in monthly rainfall were examined by comparing monthly values of the parameters (malaria cases, rainfall, time series data) obtained during the study period (9 years).

The significance level of all comparison was set at 0.05. All reported p-values are two-tailed.

## Results

### Prevalence of febrile cases

During the nine-year study period, slides from 28,092 febrile children were examined at the MCRU. The number of children screened showed two peaks, in 2002 (n = 4867) and in 2008 (n = 4684). There was a decrease in the yearly number of consultations for fever from 2002 to 2005, followed by a significant increase until 2008 (*p *< 0.01, Cochran-Armitage test for trend) (Table 1).

**Table 1:** Proportion of malaria cases according to age (between 2000 to 2008)

	2000	2001	2002	2003	2004	2005	2006	2007	2008
**Number of children**	889	3525	4867	4133	2932	1174	2172	3116	4684
**Age, Median(IQR)^**a**^, months**	24(13-48)	24(12-48)	24(13-54)	24(12-48)	24(12-54)	24(12-48)	24(12-48)	24(12-60)	24(12-48)
**Proportion with Malaria**	45%	27%	36.7%	36.4%	30.0%	24.5%	21.0%	12.0%	15.0%
**Children (0- <5 years), N(%)**	699 (79%)	2795 (79%)	3656 (75%)	3159 (76%)	2190 (75%)	1347 (75%)	1655 (76%)	2327 (75%)	3630 (78%)
-proportion with malaria	45%	26%	36%	34%	28%	21%	19%	10%	13%
**Children (5-10 years), N,(%)**	190 (21%)	730 (22%)	1211 (25%)	974 (24%)	742 (25%)	427 (25%)	517 (24%)	789 (25%)	1054 (23%)
- proportion with malaria	46%	31%	40%	43%	37%	35%	27%	16%	23%
**OR (95%CI)^b ^for malaria risk**									
- children (0 - <5 years)	Reference	Reference	Reference	Reference	Reference	Reference	Reference	Reference	Reference
- children (5-10 years)	1.0 (0.7-1.4)	1.3 (1.1-1.5)	1.2 (1.1-1.3)	1.5 (1.3-1.7)	1.6 (1.3-1.8)	2.0 (1.6-2.6)	1.5 (1.2-1.9)	1.7 (1.4-2.2)	2.0 (1.6-2.3)
*p*-value	0.91	0.004	0.02	< 0.001	< 0.001	< 0.001	< 0.001	< 0.001	< 0.001

^a^: interquartile ranges (25^th ^and 75^th ^percentiles); ^b^: Children less than five years served as reference; N: number

The median (IQR) age of children consulting for fever was globally of 24(12-48) months. This did not significantly change from one year to another over the nine years (Table 1). Overall, children younger than five years represented 76% of the screened patients; this proportion was nearly the same over the surveillance period (Table 1). More than 55% of the febrile patients screened each year at MCRU were aged 0 to 35 months.

### Malaria prevalence

There was a linear decrease in the annual number of malaria cases, from 1,785 in 2002 to 359 in 2007 with a slight increase in 2008 (*p *< 0.01, Cochran-Armitage test for trend). In 2000, the proportion of febrile illness due to malaria was 45%; this proportion declined to 12% in 2007 and 15% in 2008.

### Age and malaria

**Figure 1:**
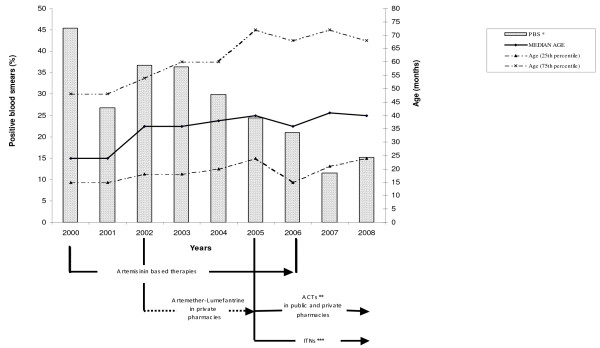
**PBS: proportion of malaria positive blood smears**.

**Figure 2:**
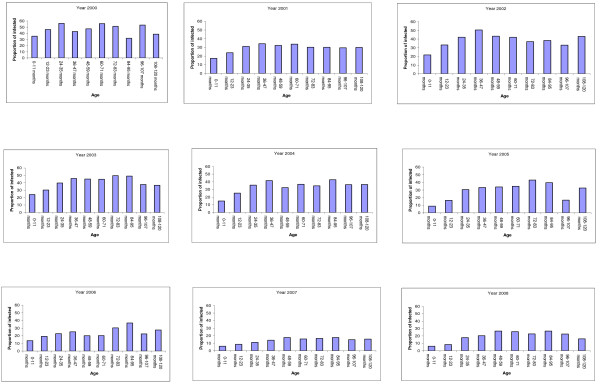
**Age distribution of the proportion of malaria cases by year among children under 11 years (from 2000 to 2008)**.

The median (IQR) age of children with a positive blood smear significantly increased from 24(15-48) to 41(21-72) months over the study period (*p *< 0.01). In 2000, parasite prevalence was highest and the median age of patients with positive slides the lowest (Figure 1). The median age of patients with malaria negative slides did not fluctuate (24(12-48)) over the time. The lowest prevalence of malaria throughout the study period was found among the 0-23 months old patients (Figure 2). The proportion of malaria cases in the febrile children increased with age until 47 months (*p *< 0.01, Cochran-Armitage test for trend); from four years old, the proportion of malaria cases was similar in the different age groups (Figure 2).

The study population was grouped into those less than five years and the oldest (Table 1). In children younger than five years old, the prevalence of malaria decreased significantly in 2001 (26%), compared to the year 2000 (*p *< 0.01). A relative increase in malaria cases of 10% was observed between 2001 and 2002, followed by a drop of about two third in 2007 (10%) and 2008 (13%) (*p *< 0.01, Cochran-Armitage test for trend). In older children (5-10 years), the proportion of malaria cases was significantly higher compared to the youngest; the risk of having malaria was 1.2 to 2.0 in older patients than younger ones (Table 1).

According to the paediatric ward, data were compared between inpatients group (n = 15299) and outpatients group (n = 7502); origin was not known for 5291 children. Between 2000 and 2005, the proportion of malaria cases among children aged less than 24 months was always higher in the inpatients group (25-35%) compared to the outpatients (11-23%) (*p *< 0.01). From 2006, the same difference was only observed for patients aged 96 to 120 months old ((*p *< 0.02). During the study period, the median age did not significantly vary among the infected inpatients, ranging from 18 to 24 months old; whereas in the infected outpatients group, it ranged from 21 to 40 months (*p *< 0.01)

### Parasite densities

Parasitaemia ranged from 14/μL to 1,611,408/μL. The GMPD did not vary significantly over the study period, and ranged from 6,800 p/μL in 2000 to 7,512 p/μL in 2008. Depending on the age, the GMPD was slightly higher in children less than five years old (6751/μL to 9,779/μL) than in children aged 5-10 years old (3,593/μL to 6,951/μL). No difference was observed when comparing GMPD between inpatient and outpatient groups (results not shown).

### Rainfall and malaria prevalence

**Figure 3:**
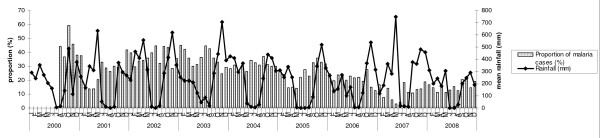
**Monthly distribution of the proportion of malaria positive blood smears and mean rainfall**.

Rainfall patterns were similar throughout the study period. June, July and August were the driest months with recorded rainfalls of 0 to 85 mm, except for the years 2000 and 2006 (Figure 3). The rest of the months were generally rainy with the wettest period from October to December (330-400 mm).

Monthly malaria prevalence data showed that there was a slight decrease in the proportion of positive slides from February to May, with a continuous increase between May and October (Figure 3). However, a comparison of the proportions of malaria cases during the rainy (mean prevalence obtained between September and May) and the dry season (mean prevalence obtained between June and August) did not show any difference over the study period. Indeed, analysis of time series auto-correlation on de-seasonalized data showed that there was an absence of correlation between means of monthly rainfall, with an autocorrelation coefficient of 0.48 (95%CI: -1.81-0.86) (standard error 0.68) (*p *= 0.48). Rainfall had no effect on the intercept of the trends in the proportion of malaria cases observed during the study period (Table 2).

**Table 2:** Results of time series analysis

Parameters	Coefficient	SE	t-stat	*P-value*	95%CI	
**Intercept**	26.84	17.22	1.56	0.12	-6.9	60.60
**Non malaria cases**	0.24	0.04	5.96	< 0.01	0.16	0.32
**Rainfall**	0.67	1.90	-0.35	0.73	-4.39	3.06

SE: standard error

## Discussion

Understanding the local epidemiology of malaria is essential to design the best methods to control the disease. This prospective study assessed the evolution of malaria morbidity in an urban hospital care seeking febrile children of Gabon, and showed that the pattern of *P. falciparum *infection has changed from 2000 to 2008. The proportion of malaria positive slides among febrile children seen at hospital strongly declined and was accompanied by a rise in the age of children with malaria. This follows a twenty-year period during which the prevalence of malaria was 29% in 1980, and had increased to more than 40% in 2000 [18]. The evolution of the proportion of malaria cases, diagnosed using the same standardized method, did not follow the yearly recorded number of febrile patients; whatever the number of children seen at MCRU, the number of malaria cases consistently decreased over the time. The fact that the proportion of non malaria cases significantly rose during the study period while a significant downward trends in malaria cases was observed, suggests that this decline is specific to malaria.

The decrease in the prevalence of malaria started in 2001, probably due to several factors. Climatic data were analysed; rainfall patterns remained constant throughout the nine years of observation and had no impact on the drastic reduction in malaria cases. The proportion of malaria positive blood smears did not show any seasonal variation. Numerous interventions occurred in the country during the study period. Indeed, at the beginning of the study, chloroquine was the first line and the main treatment for mild malaria, despite a well documented resistance above 90% [13,14]. Consecutively, a change in malaria treatment behaviour was observed; between 2000 and 2006, artemisinin derivatives based monotherapies and artemether-lumefantrine combination (from 2002), that were widely available in the country, became the most frequently antimalarials prescribed. In 2003, artesunate-amodiaquine and artemether-lumefantrine were chosen as first and second line treatments for uncomplicated malaria [15]. This policy decision, supported by the Global Fund, led to the deployment of free distribution of ACTs and ITNs in 2005 in public health facilities, targeting children younger than five years old. Bednet impregnation campaigns began at the same time. ITNs coverage reached 49.8% in 2006 and 57% in 2008 [[1], Malaria control program, unpublished data]. More than two millions of ACTs packs have been distributed in public health centres from 2006 to 2008. This can be surprising when considering that the target population comprised 272,000 children less than five years; but presumptive treatment of fever with an antimalarial drug is still frequent in Gabon, and ACTs are freely provided to the patients, irrespective of their age [19].

The good efficacy of artemisinin derivatives based therapies, which was evaluated to be higher than 95% in 2005, and the use of ITNs may have contributed, independently or in association, to the reduction of the morbidity of the disease as demonstrated elsewhere [8,20-23].

Throughout the nine-year study period, the burden of febrile illness remained highest among children under five years old. However, the median age of children with malaria significantly increased, as the prevalence of the infection declined. From 2001, children over five years of age were more likely to have malaria, suggesting a higher exposure (though that most of them do not benefit from free ITNs in Libreville) or a delayed acquisition of immunity, as observed by other authors [24-27]. However, this changing age pattern was not observed in the inpatient group, then, the severity of the disease remained confined to the youngest as reported elsewhere [3,6,28].

The decline of malaria prevalence and the age shift towards the older children may indicate a change in the dynamics of malaria epidemiology in Libreville. It has been reported that the age at which patients experience malaria increases with a decline in exposure, and may precede changes in malaria transmission [3,7,25]. Furthermore, urbanization is extending in Libreville, creating a hostile environment to anophelinae vector development.

This study was a health facility-based survey, probably not representative of the situation in the entire country; in several private health centres and rural areas, laboratory and/or well-trained microscopists are lacking. Moreover, the function of the MCRU in the CHL is to provide results of freely available microscopic diagnosis of malaria to clinicians working in the different wards, information on malaria clinical presentations, outcomes and treatments are missing.

## Conclusion

The proportion of malaria among febrile children has decreased during the last nine years, probably due to the impact of the utilization of more effective antimalarial drugs, artemisinin derivatives based therapies. After the wide-scale introduction of new effective prevention and disease management strategies, this decline was more pronounced. All these interventions are likely to be the main possible contributing factors to the observed malaria changes. Preventive measures must be further implemented and extended to older children. Studies on the prevalence of malaria in other parts of the country and record of entomological data are needed to characterize the transmission levels in Gabon.

## Competing interests

The authors declare that they have no competing interests.

## Authors' contributions

The study was initiated by MK and PGK; and conducted by MK with contribution of all authors. MKBA and DPMM interpreted the data and drafted the manuscript. Data were analysed by MKBA and EK. MMM, EBN, TP, ADE, MPM, EI, EEE and MCRU team participated to the field study. All authors revised and approved the final manuscript.
